# A second triclinic polymorph of (1-ammonio-1-phosphono­eth­yl)phospho­nate

**DOI:** 10.1107/S1600536811022239

**Published:** 2011-06-18

**Authors:** Natalia V. Tsaryk, Anatolij V. Dudko, Alexandra N. Kozachkova, Vasily I. Pekhnyo

**Affiliations:** a Institute of General and Inorganic Chemistry, National Academy of Science Ukraine, Prospekt Palladina 32/34, Kyiv 03680, Ukraine

## Abstract

The asymmetric unit of the second polymorph of the title compound, C_2_H_9_NO_6_P_2_, contains one mol­ecule existing as a zwitterion. The N atom of the ammonio group is protonated and one of the phospho­nic acid groups is deprotonated. Bond lengths and angles are similar in both polymorphs. Besides the differences in cell parameters, the most significant structural difference between this structure and that of the first polymorph [Dudko, Bon, Kozachkova, Tsarik & Pekhno (2008 ▶), *Ukr. Khim. Zh.* **74**, 104–106] is the presence of strong symmetric hydrogen bonds between neighbouring phospho­nate groups. H atoms involved in these hydrogen bonds are located at inversion centres and O⋯O distances are observed in the range 2.458 (5)–2.523 (5) Å. These bonds and additional O—H⋯O and N—H⋯O hydrogen bonds inter­link the mol­ecules, giving a three-dimensional supromolecular network.

## Related literature

For the original polymorph, see: Dudko *et al.* (2008 ▶). For similar bis­phospho­nates, see: Fernández *et al.* (2003 ▶); Li *et al.* (2009 ▶). For general background on the usage of organic diphospho­nic acids as chelating agents in metal extraction and as drugs to prevent calcification and inhibit bone resorption, see: Matczak-Jon & Videnova-Adrabinska (2005 ▶); Matkovskaya *et al.* (2001 ▶). For examples of symmetrical O—H⋯O hydrogen bonds, see Catti & Ferraris (1976 ▶); Meot-Ner (2005 ▶). For bond-length data, see: Allen *et al.* (1987 ▶).
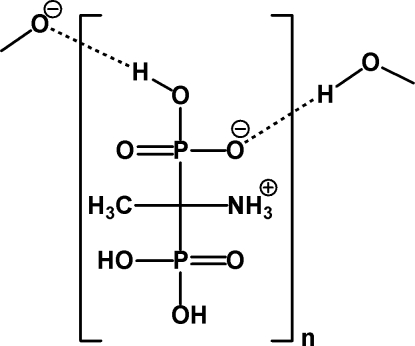


## Experimental

### 

#### Crystal data

C_2_H_9_NO_6_P_2_
*M* *_r_* = 205.04
Triclinic, 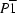

*a* = 5.5674 (11) Å
*b* = 5.9023 (12) Å
*c* = 11.385 (2) Å
α = 82.334 (10)°
β = 82.145 (9)°
γ = 78.148 (10)°
*V* = 360.56 (12) Å^3^
*Z* = 2
Mo *K*α radiation
μ = 0.59 mm^−1^
*T* = 296 K
0.56 × 0.16 × 0.09 mm

#### Data collection

Bruker APEXII CCD diffractometer
Absorption correction: multi-scan (*SADABS*; Bruker, 2007 ▶) *T*_min_ = 0.734, *T*_max_ = 0.949
4331 measured reflections
1401 independent reflections
861 reflections with *I* > 2σ(*I*)
*R*_int_ = 0.058

#### Refinement

*R*[*F* ^2^ > 2σ(*F* ^2^)] = 0.049
*wR*(*F* ^2^) = 0.122
*S* = 1.06
1401 reflections
116 parameters
H atoms treated by a mixture of independent and constrained refinement
Δρ_max_ = 0.45 e Å^−3^
Δρ_min_ = −0.43 e Å^−3^

### 

Data collection: *APEX2* (Bruker, 2007 ▶); cell refinement: *SAINT* (Bruker, 2007 ▶); data reduction: *SAINT*; program(s) used to solve structure: *SHELXS97* (Sheldrick, 2008 ▶); program(s) used to refine structure: *SHELXL97* (Sheldrick, 2008 ▶); molecular graphics: *SHELXTL* (Sheldrick, 2008 ▶); software used to prepare material for publication: *SHELXTL*.

## Supplementary Material

Crystal structure: contains datablock(s) I, global. DOI: 10.1107/S1600536811022239/im2293sup1.cif

Structure factors: contains datablock(s) I. DOI: 10.1107/S1600536811022239/im2293Isup2.hkl

Supplementary material file. DOI: 10.1107/S1600536811022239/im2293Isup3.cml

Additional supplementary materials:  crystallographic information; 3D view; checkCIF report

## Figures and Tables

**Table 1:** Hydrogen-bond geometry (Å, °)

*D*—H⋯*A*	*D*—H	H⋯*A*	*D*⋯*A*	*D*—H⋯*A*
O1—H1O⋯O2^i^	0.81 (6)	1.70 (6)	2.507 (5)	170 (6)
O3—H3O⋯O4^ii^	0.85 (7)	1.63 (7)	2.475 (5)	175 (7)
O5—H5O⋯O5^iii^	1.23 (1)	1.23 (1)	2.458 (5)	180 (0)
O6—H6O⋯O6^iv^	1.26 (1)	1.26 (1)	2.523 (6)	180 (1)
N1—H1*A*⋯O2^v^	0.90 (6)	2.11 (6)	2.929 (6)	150 (5)
N1—H1*B*⋯O4^v^	0.86 (6)	2.06 (6)	2.896 (5)	165 (5)
N1—H1*C*⋯O6^ii^	0.82 (6)	2.50 (6)	3.196 (6)	145 (5)

Symmetry codes: (i) 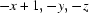; (ii) 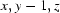; (iii) 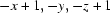; (iv) 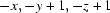; (v) 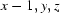.

**Fig. 1.**
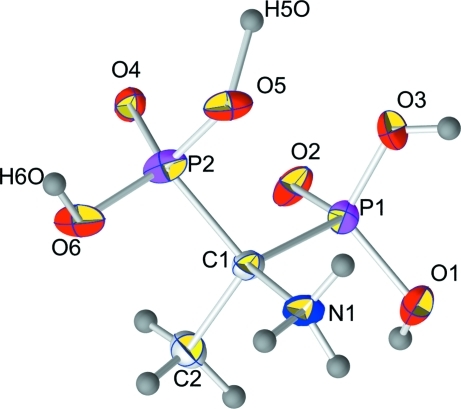
View of the molecular structure of the title compound. Ellipsoids are drawn at the 50% probability level. H atoms are represented as small spheres of arbitrary radii.

**Fig. 2.**
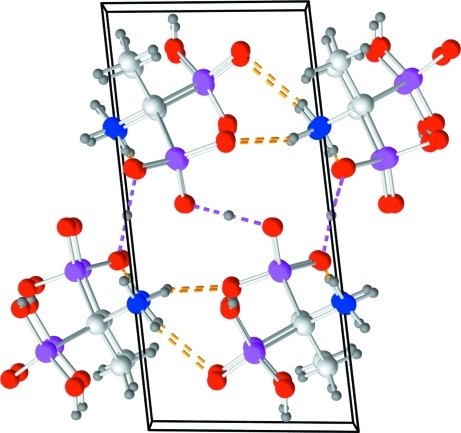
Crystal packing of title compound, projection along b axis. Dashed pink lines indicate symmetrical hydrogen bonds while dashed yellow lines indicate ordinary hydrogen bonds.

## supplementary crystallographic information

### Comment

Diphosphonic acids with the P–C–P fragment, commonly named bisphosphonates, are well known since the 19^th^ century. Due to the specific geometry and mutual influence of phosphonate fragments, compounds of this class possess a number of unique properties compared to other derivatives of phosphonic acids. Diphosphonic acids are structural analogues of inorganic pyrophosphate, one of the major metabolites in the cells, involved as a product in more than sixty biochemical reactions. Drugs prepared on the basis of bisphosphonates are highly efficient as a regulator of calcium metabolism and the immune response, they are used as anti-neoplastic, anti-inflammatory and antiviral agents and drugs with analgesic effect and as a component of toothpastes biphosphonates prevent the formation of tartar (Matkovskaya *et al.*, 2001).

However, it is still not clearly understood why small structural modifications of the bisphosphonates may lead to extensive alterations in their physicochemical, biological and toxicological characteristics (Matczak-Jon & Videnova-Adrabinska, 2005). As a consequence of that determination of the structure of the bisphosphonates is very important to understand the influence of structural modifications on complex-forming abilities and physiological activities and deriving structure properties relations in general. In the present work we report a second polymorph of the title compound which crystallizes in the space group (*P*1) whereas the previously described polymorph modification also crystallizes in the triclinic space group *P*1 but with different cell parameters and cell volume (Dudko *et al.*, 2008). The asymmetric unit of the title compound (Fig. 1) contains one molecule in zwitterionic form with a proton transferred from one of the phosphonic groups to the amino group which is typical for all investigated 1-aminodiphosphonic acids (Fernández *et al.*, 2003; Li *et al.*, 2009). Bond lengths and angles are within normal ranges (Allen *et al.*, 1987) and are comparable with the first polymorph modification of the compound. It is generally accepted that, for O—H—O interactions where O···O is about 2.50 Å, examples can be found of truly symmetric hydrogen bonds, most of which have crystallographic equivalence between donor–acceptor atoms (Meot-Ner, 2005; Catti & Ferraris, 1976). The title compound, displays such short and strong symmetric hydrogen bonds between neighboring phosphonate groups, with the H atoms located at inversion centres and O···O distances of 2.458 (5) and 2,523 (5) Å. Multiple N—H···O and O—H···O hydrogen bonds in the crystal structure form an intricate three-dimensional supramolecular network (Fig.2, Table 1).

### Experimental

The title compound C_2_H_9_NO_6_P_2_, was obtained by the reaction of acetonitrile which was brought into contact with the dry hydrogen chloride and phosphorus trichloride at 278 K followed by the dropwise addition of water. The obtained solution was treated by a mixture of acetone and diethyl ether, resulting in a white precipitate of the title compound. The resulting residue was dissolved in water and was stored in a dark place for slow evaporation. After 14 d of staying, suitable crystals for X-ray data collection were obtained.

### Refinement

H atoms bonded to O and N atoms were located in a difference Fourier map. Their positions were refined freely whereas thermal parameters were fixed to *U*_iso_(H) = 1.5*U*_eq_(N,O). Other H atoms which bonded to C were positioned geometrically and refined using a riding model with C—H = 0.96 Å for CH_3_ with *U*_iso_(H) = 1.5*U*_eq_(C).

### Crystal data

**Table:**

C_2_H_9_NO_6_P_2_	*Z* = 2
*M**_r_* = 205.04	*F*(000) = 212
Triclinic, *P*1	*D*_x_ = 1.889 Mg m^−^^3^
Hall symbol: -P 1	Melting point: 551 K
*a* = 5.5674 (11) Å	Mo *K*α radiation, λ = 0.71073 Å
*b* = 5.9023 (12) Å	Cell parameters from 1315 reflections
*c* = 11.385 (2) Å	θ = 3.6–25.3°
α = 82.334 (10)°	µ = 0.59 mm^−^^1^
β = 82.145 (9)°	*T* = 296 K
γ = 78.148 (10)°	Needle, colourless
*V* = 360.56 (12) Å^3^	0.56 × 0.16 × 0.09 mm

### Data collection

**Table:**

Bruker APEXII CCD diffractometer	1401 independent reflections
Radiation source: fine-focus sealed tube	861 reflections with *I* > 2σ(*I*)
graphite	*R*_int_ = 0.058
φ and ω scans	θ_max_ = 26.0°, θ_min_ = 1.8°
Absorption correction: multi-scan (*SADABS*; Bruker, 2007)	*h* = −6→6
*T*_min_ = 0.734, *T*_max_ = 0.949	*k* = −7→7
4331 measured reflections	*l* = −12→14

### Refinement

**Table:**

Refinement on *F*^2^	Primary atom site location: structure-invariant direct methods
Least-squares matrix: full	Secondary atom site location: difference Fourier map
*R*[*F*^2^ > 2σ(*F*^2^)] = 0.049	Hydrogen site location: inferred from neighbouring sites
*wR*(*F*^2^) = 0.122	H atoms treated by a mixture of independent and constrained refinement
*S* = 1.06	*w* = 1/[σ^2^(*F*_o_^2^) + (0.0387*P*)^2^ + 0.6717*P*] where *P* = (*F*_o_^2^ + 2*F*_c_^2^)/3
1401 reflections	(Δ/σ)_max_ < 0.001
116 parameters	Δρ_max_ = 0.45 e Å^−^^3^
0 restraints	Δρ_min_ = −0.43 e Å^−^^3^

### Special details

**Table:**

Geometry. All e.s.d.'s (except the e.s.d. in the dihedral angle between two l.s. planes) are estimated using the full covariance matrix. The cell e.s.d.'s are taken into account individually in the estimation of e.s.d.'s in distances, angles and torsion angles; correlations between e.s.d.'s in cell parameters are only used when they are defined by crystal symmetry. An approximate (isotropic) treatment of cell e.s.d.'s is used for estimating e.s.d.'s involving l.s. planes.
Refinement. Refinement of *F*^2^ against ALL reflections. The weighted *R*-factor *wR* and goodness of fit *S* are based on *F*^2^, conventional *R*-factors *R* are based on *F*, with *F* set to zero for negative *F*^2^. The threshold expression of *F*^2^ > σ(*F*^2^) is used only for calculating *R*-factors(gt) *etc*. and is not relevant to the choice of reflections for refinement. *R*-factors based on *F*^2^ are statistically about twice as large as those based on *F*, and *R*-factors based on ALL data will be even larger.

### Fractional atomic coordinates and isotropic or equivalent isotropic displacement parameters (Å^2^)

**Table:**

	*x*	*y*	*z*	*U*_iso_*/*U*_eq_	
P1	0.4512 (2)	−0.0726 (2)	0.18108 (11)	0.0243 (4)	
P2	0.2798 (2)	0.2733 (2)	0.36756 (11)	0.0266 (4)	
O1	0.3269 (6)	−0.1949 (6)	0.1024 (3)	0.0327 (9)	
H1O	0.337 (11)	−0.133 (11)	0.034 (5)	0.049*	
O2	0.6470 (5)	0.0492 (6)	0.1152 (3)	0.0337 (9)	
O3	0.5438 (7)	−0.2366 (6)	0.2838 (4)	0.0475 (11)	
H3O	0.531 (12)	−0.379 (12)	0.301 (6)	0.071*	
O4	0.5260 (5)	0.3445 (5)	0.3268 (3)	0.0236 (8)	
O5	0.2923 (6)	0.0865 (7)	0.4724 (3)	0.0344 (9)	
H5O	0.5000	0.0000	0.5000	0.052*	
O6	0.0723 (6)	0.4792 (7)	0.3914 (3)	0.0421 (10)	
H6O	0.0000	0.5000	0.5000	0.063*	
N1	−0.0104 (8)	0.0192 (9)	0.2928 (4)	0.0287 (11)	
H1A	−0.086 (10)	−0.025 (9)	0.237 (5)	0.043*	
H1B	−0.151 (10)	0.102 (10)	0.316 (5)	0.043*	
H1C	0.030 (10)	−0.091 (10)	0.342 (5)	0.043*	
C1	0.1979 (8)	0.1433 (8)	0.2439 (4)	0.0195 (10)	
C2	0.1069 (9)	0.3297 (8)	0.1441 (4)	0.0278 (12)	
H2A	0.0827	0.2556	0.0776	0.042*	
H2B	0.2276	0.4265	0.1191	0.042*	
H2C	−0.0464	0.4235	0.1730	0.042*	

### Atomic displacement parameters (Å^2^)

**Table:**

	*U*^11^	*U*^22^	*U*^33^	*U*^12^	*U*^13^	*U*^23^
P1	0.0236 (7)	0.0195 (7)	0.0316 (7)	−0.0033 (5)	−0.0072 (6)	−0.0066 (6)
P2	0.0204 (7)	0.0387 (9)	0.0230 (7)	−0.0080 (6)	−0.0030 (5)	−0.0074 (6)
O1	0.038 (2)	0.028 (2)	0.037 (2)	−0.0124 (17)	−0.0077 (18)	−0.0095 (17)
O2	0.0203 (18)	0.045 (2)	0.042 (2)	−0.0155 (16)	0.0069 (16)	−0.0208 (18)
O3	0.065 (3)	0.016 (2)	0.068 (3)	−0.0036 (19)	−0.042 (2)	0.002 (2)
O4	0.0182 (16)	0.0200 (18)	0.0344 (19)	−0.0022 (13)	−0.0056 (14)	−0.0091 (15)
O5	0.0272 (19)	0.055 (2)	0.0241 (18)	−0.0172 (17)	−0.0077 (15)	0.0035 (17)
O6	0.0231 (19)	0.067 (3)	0.034 (2)	0.0124 (18)	−0.0057 (16)	−0.0270 (19)
N1	0.017 (2)	0.036 (3)	0.033 (3)	−0.009 (2)	−0.006 (2)	0.005 (2)
C1	0.016 (2)	0.022 (3)	0.022 (2)	−0.0064 (19)	−0.0040 (19)	0.000 (2)
C2	0.031 (3)	0.023 (3)	0.027 (3)	0.000 (2)	−0.007 (2)	−0.001 (2)

### Geometric parameters (Å, °)

**Table:**

P1—O2	1.490 (3)	O5—H5O	1.229 (0)
P1—O3	1.493 (4)	O6—H6O	1.261 (0)
P1—O1	1.533 (4)	N1—C1	1.502 (6)
P1—C1	1.831 (5)	N1—H1A	0.90 (6)
P2—O4	1.510 (3)	N1—H1B	0.86 (6)
P2—O5	1.512 (4)	N1—H1C	0.82 (6)
P2—O6	1.520 (3)	C1—C2	1.534 (6)
P2—C1	1.840 (4)	C2—H2A	0.9600
O1—H1O	0.81 (6)	C2—H2B	0.9600
O3—H3O	0.85 (7)	C2—H2C	0.9600
			
O2—P1—O3	111.7 (2)	C1—N1—H1B	118 (4)
O2—P1—O1	114.3 (2)	H1A—N1—H1B	87 (4)
O3—P1—O1	110.8 (2)	C1—N1—H1C	113 (4)
O2—P1—C1	109.0 (2)	H1A—N1—H1C	110 (5)
O3—P1—C1	106.5 (2)	H1B—N1—H1C	111 (6)
O1—P1—C1	103.86 (19)	N1—C1—C2	107.8 (4)
O4—P2—O5	112.46 (18)	N1—C1—P1	107.2 (3)
O4—P2—O6	112.8 (2)	C2—C1—P1	109.4 (3)
O5—P2—O6	111.7 (2)	N1—C1—P2	107.4 (3)
O4—P2—C1	107.07 (18)	C2—C1—P2	111.7 (3)
O5—P2—C1	106.2 (2)	P1—C1—P2	113.1 (2)
O6—P2—C1	105.98 (19)	C1—C2—H2A	109.5
P1—O1—H1O	110 (4)	C1—C2—H2B	109.5
P1—O3—H3O	128 (4)	H2A—C2—H2B	109.5
P2—O5—H5O	115.9 (0)	C1—C2—H2C	109.5
P2—O6—H6O	115.1 (0)	H2A—C2—H2C	109.5
C1—N1—H1A	115 (3)	H2B—C2—H2C	109.5
			
O2—P1—C1—N1	171.5 (3)	O4—P2—C1—N1	166.1 (3)
O3—P1—C1—N1	−67.9 (3)	O5—P2—C1—N1	45.8 (3)
O1—P1—C1—N1	49.2 (3)	O6—P2—C1—N1	−73.2 (4)
O2—P1—C1—C2	54.9 (4)	O4—P2—C1—C2	−76.0 (3)
O3—P1—C1—C2	175.5 (3)	O5—P2—C1—C2	163.7 (3)
O1—P1—C1—C2	−67.4 (3)	O6—P2—C1—C2	44.7 (4)
O2—P1—C1—P2	−70.3 (3)	O4—P2—C1—P1	48.0 (3)
O3—P1—C1—P2	50.3 (3)	O5—P2—C1—P1	−72.4 (3)
O1—P1—C1—P2	167.4 (2)	O6—P2—C1—P1	168.7 (2)

### Hydrogen-bond geometry (Å, °)

**Table:**

*D*—H···*A*	*D*—H	H···*A*	*D*···*A*	*D*—H···*A*
O1—H1O···O2^i^	0.81 (6)	1.70 (6)	2.507 (5)	170 (6)
O3—H3O···O4^ii^	0.85 (7)	1.63 (7)	2.475 (5)	175 (7)
O5—H5O···O5^iii^	1.23 (1)	1.23 (1)	2.458 (5)	180 (0)
O6—H6O···O6^iv^	1.26 (1)	1.26 (1)	2.523 (6)	180.(1)
N1—H1A···O2^v^	0.90 (6)	2.11 (6)	2.929 (6)	150 (5)
N1—H1B···O4^v^	0.86 (6)	2.06 (6)	2.896 (5)	165 (5)
N1—H1C···O6^ii^	0.82 (6)	2.50 (6)	3.196 (6)	145 (5)

Symmetry codes: (i) −*x*+1, −*y*, −*z*; (ii) *x*, *y*−1, *z*; (iii) −*x*+1, −*y*, −*z*+1; (iv) −*x*, −*y*+1, −*z*+1; (v) *x*−1, *y*, *z*.
